# Recent Advances in High-Throughput Nanomaterial Manufacturing for Hybrid Flexible Bioelectronics

**DOI:** 10.3390/ma14112973

**Published:** 2021-05-31

**Authors:** Nathan Zavanelli, Jihoon Kim, Woon-Hong Yeo

**Affiliations:** 1 George W. Woodruff School of Mechanical Engineering, Center for Human-Centric Interfaces and Engineering at the Institute for Electronics and Nanotechnology, Georgia Institute of Technology, Atlanta, GA 30332, USA; nzavanelli@gatech.edu (N.Z.); louisjihoonkim@gatech.edu (J.K.); 2 Wallace H. Coulter Department of Biomedical Engineering, Georgia Institute of Technology, Atlanta, GA 30332, USA; 3 Parker H. Petit Institute for Bioengineering and Biosciences, Neural Engineering Center, Institute for Materials, Institute for Robotics and Intelligent Machines, Georgia Institute of Technology, Atlanta, GA 30332, USA

**Keywords:** nanomanufacturing, high-throughput method, material printing, flexible bioelectronics

## Abstract

Hybrid flexible bioelectronic systems refer to integrated soft biosensing platforms with tremendous clinical impact. In this new paradigm, electrical systems can stretch and deform with the skin while previously hidden physiological signals can be continuously recorded. However, hybrid flexible bioelectronics will not receive wide clinical adoption until these systems can be manufactured at industrial scales cost-effectively. Therefore, new manufacturing approaches must be discovered and studied under the same innovative spirit that led to the adoption of novel materials and soft structures. Recent works have taken mature manufacturing approaches from the graphics industry, such as gravure, flexography, screen, and inkjet printing, and applied them to fully printed bioelectronics. These applications require the cohesive study of many disparate parts. For instance, nanomaterials with optimal properties for each specific application must be dispersed in printable inks with rheology suited to each printing method. This review summarizes recent advances in printing technologies, key nanomaterials, and applications of the manufactured hybrid bioelectronics. We also discuss the existing challenges of the available nanomanufacturing methods and the areas that need immediate technological improvements.

## 1. Introduction

There is a fundamental mismatch between biological systems, which are soft and deformable, and traditional electronics, which are rigid and impermeable to sweat and liquids [1,2,3,4,5]. This incongruity places a significant constraint on the development of bioelectronics systems [4,5]. Traditional systems cannot conform well to the human body, making most wearable devices susceptible to large noise during motion, uncomfortable and obtrusive to wear, and limited to very specific regions on the body, such as the wrist, chest, and finger, that allow for easy attachment of rigid systems [1,3]. Furthermore, rigid implantable electronics and surgical instruments cannot easily integrate with the soft systems for which they are targeted [2]. As a result, current applications of wearable electronics, biosensors, and implantable healthcare are highly limited, leaving millions of people with serious undiagnosed diseases and allowing the steady progression towards heart attack and stroke to remain undetected [2,5]. In contrast, recent advances in hybrid electronics have yielded new classes of electronic devices and sensors that integrate well with the human body [6,7,8,9]. These systems can achieve high stretchability or flexibility through two paradigms: first, metal depositions on the order of 10 µm or less can easily bend in accordance with Euler–Bernoulli theory because of their minimal height, and fractal geometric patterns can be introduced to allow for stretching with minimal local strain as the patterns unfold [10]. Second, conductive nanomaterial–polymer matrices can be made intrinsically stretchable by maintaining conductive pathways as the polymer undergoes strain [11]. Both novel systems can stretch and deform when needed, allowing for seamless integration with the skin [8]. As a result, previously inaccessible physiological signals and implantable healthcare targets can be realized, promising a transformation in modern medicine [7]. Likewise, traditional biosensing methods, such as florescent microarrays, lateral flow immunoassays, DNA microarrays, enzyme-linked immunosorbent assays, and polymerase chain reaction-based methods require expensive reagents and laboratory equipment, and are limited by slow signal processing methods; however, fully printed sensors allow for real time, continuous biomarker quantification in a simple, affordable, and mass producible package [12,13]. Printed biomolecule sensors promise a transformative way to continuously assess crucial biomarkers, making their development critical in the future of healthcare development [12,14,15,16]. These systems were initially fabricated with traditional micro- and nano-electromechanical systems (MEMS/NEMS) approaches, but such techniques are poorly suited for the industrial scales necessary to commercialize hybrid electronics. For hybrid bioelectronics to achieve their potential, the same innovative spirit that led to the adoption of new materials must be applied to the study of new manufacturing approaches.

Fully printed electronics methods have gained significant interest in recent years because they are cheaper, more efficient, and more scalable than traditional MEMS/NEMS processes and capable of direct printing on many flexible and stretchable substrates, such as polyethylene terephthalate (PET), polyimide (PI), polydimethylsiloxane (PDMS), thermoplastic polyurethane (TPU) and paper [17,18,19]. However, these approaches are also limited by product throughputs and yields, making it very difficult to manufacture hybrid devices in a manner that would allow for their mass adoption [17]. For instance, aerosol jet printing has demonstrated very high print resolutions and control over material heights and microarchitectures, but the additive deposition of numerous nano thickness layers makes it by necessity a low-speed manufacturing option [20]. Inkjet printing is another attractive printing method, but it is limited by the requirement that particles are small and well dispersed enough not to clog the inkjet nozzle, and traditional inkjet printing is also too low throughput for industrial scales [17,21]. However, roll-to-roll inkjet printing has been demonstrated, making it a potential target for high-speed device manufacturing [22]. Electrohydrodynamic printing is an exciting new method that can overcome several of the key challenges in inkjet printing by pulling ionized inks directly to the substrate, but it is likewise limited by constrictive stand-off height requirements and low manufacturing yields [21]. In contrast, contact printing methods, such as screen, gravure, flexographic, slot die, and doctor blade printing are easily integrated with high throughput roll to roll systems, making them an excellent option for industrial scale hybrid bioelectronics manufacturing. A summary of each of the high throughput nanomaterial fabrication methods discussed in this review is provided in Table 1 [17,23,24,25,26,27,28,29,30,31,32].

**Figure 1:**
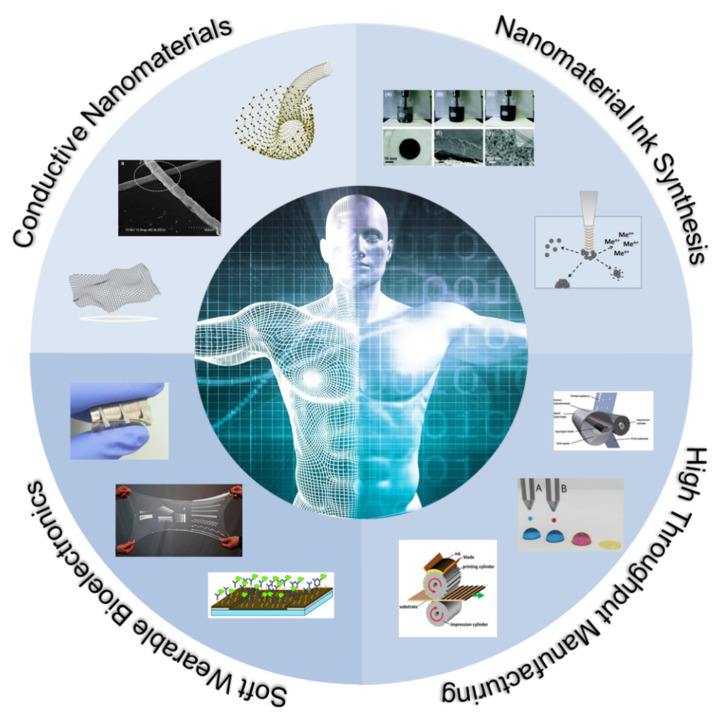
Overview of high-throughput nanomaterial fabrication methods and applications in soft bioelectronics. (Figures are adapted or reprinted, clockwise from the top: (1) *RSC Adv.* (2013), 3, 24812, Copyright 2013, RSC, (2) *J. Nanoparticle Res.* (2016), 18, 285, Copyright 2106, Springer, (3,7) *Ind. Eng. Chem. Res.* (2019), 58, 43, 19909–19916, Copyright 2019, ACS, (4) *Adv. Mater. Interfaces* (2018), 5, 1701561. Copyright 2018, Wiley, (5) *Adv. Mater.* (2019), 31, 1806702. Copyright 2020, Wiley, (6) Creative Commons License CC BY from *J. Electrochem. Soc*. (2018), 165 B3084, (8) *ACS Appl. Mater. Interfaces* (2020), 12, 4146797–46803. Copyright 2020, ACS, (10) Creative Commons license CC BY from *Sci. Rep.* (2017), 7(1)).

There are four crucial design challenges that must be met to make high throughput manufacturing of hybrid bioelectronics a reality, as depicted in Figure 1 [16,33,34,35,36,37,38,39], and all four are highly interdependent on each other. First, conductive nanomaterials must be identified and produced based on the final application requirements. Second, these nanomaterials must be dispersed in a fully printable ink with rheology and viscosity well suited to the specific printing method. Third, innovative manufacturing techniques must be explored that can achieve scales suited for mass production. Finally, the printed inks must be sintered and cured after printing in a way that does not limit manufacturing speed.

In this review, we will summarize recent attempts to develop high-throughput manufacturing of nanomaterials for hybrid bioelectronics, with a specific focus on new printing methods, nanomaterial selection, synthesis, dispersion and printing, post-print processing and demonstrated applications. We will begin with a discussion on several of the key manufacturing methods under investigation, emphasizing how deposition physics leads to key constraints on ink design, print resolutions, deposition heights, device yield, and print speed and novel approaches to push the field beyond its traditional limitations. Next, we will summarize the key nanomaterials that are used in printed bioelectronics. For each material, we will summarize the key mechanical, chemical, and electrical properties that determine the material’s functionality, recent advances, and challenges in ink formulations, demonstrated bioelectronics devices fabricated with high throughput methods, and novel high throughput sintering methods. Finally, we will comment on the state-of-the-art in the field, assess the key limitations yet to be solved, and look forward to the future development of high-throughput nanomaterial manufacturing for soft bioelectronics.

## 2. Printing Fundamentals

### 2.1. Gravure Printing

**Figure 2:**
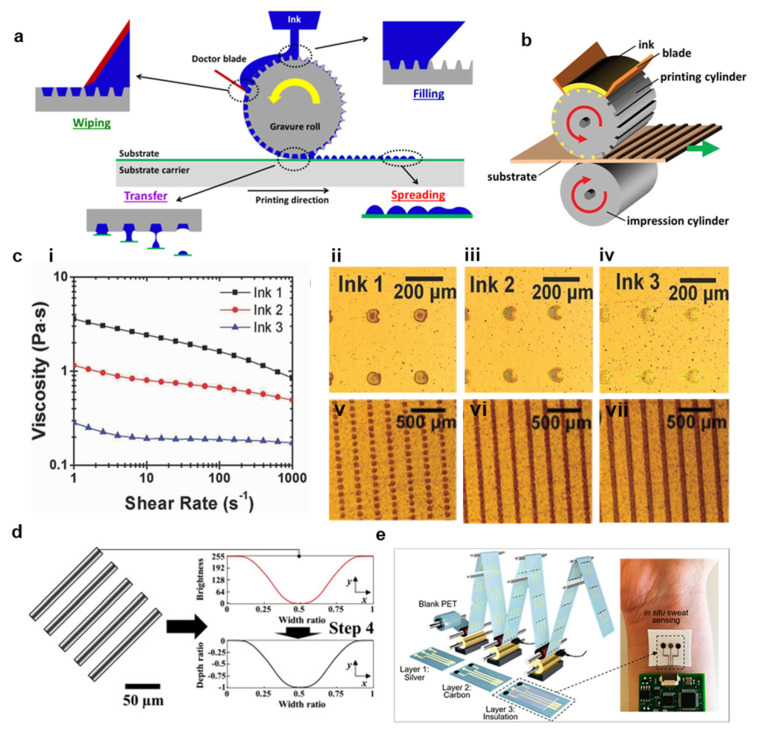
Gravure printing of hybrid bioelectronics. (**a**) Overview of the gravure printing process (reprinted with permission from *Flex. Print. Electron* (2016), 1 023003. Copyright 2016, IOP). (**b**) Illustration of gravure printing linear traces against an impression roll. (reprinted with permission from *Adv. Mater.* (2019), 31, 1806702. Copyright 2020, Wiley). (**c**) Optimizing graphene inks for gravure printing. (**i**) Characterization of viscosity for the three different ink formulations. (**ii**–**iv**) Images of printed dots for each ink using a gravure cell of 50 µm. (**v**–**vii**) Images showing line formation as the cell spacing is reduced, corresponding to 50, 25, and 5 µm spacing for a cell size of 50 µm. (reproduced with permission from *Adv. Mater.* (2014), 26: 4533–4538. Copyright 2014, Wiley). (**d**) Optimized cell patterns achieved with gradation engraving to achieve high-resolution gravure printing. (reprinted with permission from *Precis. Eng.* (2021), 69: 1–7. Copyright 2021, Elsevier). (**e**) Illustration of roll-to-roll printed sweat sensors. (reprinted with permission from *ACS Nano* (2018), 12(7): 6978–6987, Copyright 2018, ACS).

Gravure printing is a mature manufacturing method that has been employed for high throughput image printing since the 19th century [27]. Gravure printing is achieved in four phases, as shown in Figure 2a,b [27,34]. First, ink is poured on a rotating gravure roll and fills the recessed cells in the roll [40]. Second, a doctor blade removes the excess ink from the roll, leaving only an ink thickness corresponding to the depth of the cell [40]. Third, the ink is brought in contact with a substrate, which is itself being rolled at the same speed as the gravure roll, and the ink is pulled from the roll onto the substrate as a result of adhesive forces between the ink and the substrate and the ink’s surface tension [27,40]. Finally, the print will stabilize on the substrate and spread based on the theoretical contact angle that the liquid–gas interface makes to the substrate, which is determined by Young’s equation. $cos\theta_{c}=\frac{\gamma_{sg}-\gamma_{sl}}{\gamma_{lg}}$, where $\gamma_{sg}$, $\gamma_{sl}$, $\gamma_{lg}$ are the surface energies for solid–liquid, liquid-gas, and solid-gas, respectively [27,50,51]. Although this is true for all direct printing methods, it is particularly important for gravure printing because gravure patterns consist of individual cells which must spread into each other to form a cohesive print [52]. In addition, print resolution, quality, and speed are primarily limited by the complex fluid dynamics occurring when excess ink is removed by the doctor blade [51]. Printing faults during this phase are broadly characterized by two processes: lubrication residue and ink drag out [27,53]. First, the doctor blade will always leave a small residual ink layer on the roll, and this layer’s thickness must be substantially reduced to prevent electrical shorts and erroneous material depositions [27]. The doctor blade’s efficiency depends heavily on the relative magnitudes of viscous forces and surface tension. This relationship is captured in the capillary number $C_{a}=\frac{viscous\;forces}{surface\;tension}=\frac{\mu U}{\sigma }$, where *µ* is the ink viscosity, *U* is the print speed, and σ is the ink surface tension [51].

At high capillary numbers, the residual thickness is often unacceptable [27]. Therefore, reducing print speed and ink viscosity is essential in limiting lubrication residue. Second, the doctor blade may pull ink out of the cells as it passes and deposits the ink on the roll in a process termed drag out [53]. This process has been analytically and empirically shown to depend heavily on capillary flow, which is limited at high capillary numbers [51,52]. In this condition, the print velocity is too high compared to the capillary flow characteristic velocity for drag out to occur [27]. Therefore, high capillary numbers prevent drag out and low capillary numbers limit lubrication residue. In practice, achieving a capillary number of Ca ≈ 1 is necessary for high-quality gravure printing, although the ideal capillary number also depends on pattern geometry, orientation, substrate wetting, and print thickness [54]. Because these interactions are often complicated to determine analytically a priori, especially with viscoelastic inks, many researchers optimize their process with statistical design of experiments techniques, such as analysis of variance and Taguchi methods [51]. Figure 2c depicts results from one such experiment to determine the optimal ink viscosity and cell spacing for a process with a fixed speed (3 m/min) [53]. Three graphene inks were formulated with various viscosities (i), and single dots were printed (ii–iv), with the low viscosity ink (iv) producing an unlevel print with an extended residue tail [53].

In addition to optimizing the ink rheology and substrate wetting, the cell pattern is crucial in achieving high-resolution prints [55]. Printing continuous lines, which is referred to as Intaglio printing, is avoided because the drag-out effect is amplified with long prints oriented in the printing direction [56]. In order to produce high resolution, level prints, minimizing cell dimensions is critical [27,52,53]. As shown in Figure 2c, previously discussed 2.5 Pa·S ink was also printed with cell spacings of 50 µm (v), 25 µm (vi) and 5 µm (vii), and the line uniformity increased significantly with a decrease in cell spacing [53]. Further increasing resolution and quality in gravure printing is complicated, however, because traditional print head fabrication methods, such as electromechanical and laser engraving, are unable to produce cells with dimensions < 10 µm, and they are also likely to produce additional roughness on the gravure roll near the cell as a result of the engraving process [52,53]. Therefore, recent works have employed silicon microfabrication techniques to design very high-resolution gravure rolls [53]. For instance, Secor et al. used photolithography to design a silicon-based gravure roll capable of producing high resolution trace <30 µm with conductivities >10,000 S/m [53]. To further reduce trace widths, Lee et al. experimented with various cell depth profiles under the hypothesis that curved cell walls would reduce drag out [52]. As shown in Figure 2d, a curved gradient pattern was able to reduce print width by 65%, yielding a final pattern of <10 µm [52]. These recent developments in high resolution and high throughput gravure manufacturing, combined with the novel advances in printable, conductive nanomaterial inks discussed in Section 3, make them well suited to numerous bioelectronics applications, including multilayer circuit fabrication and sensor manufacturing, such as the sweat sensor demonstrated in Figure 2e [40]. However, gravure printing also presents very high startup costs, incurs high costs to prototype, places rigid requirements on ink rheology, and often requires substrate surface modifications in order to achieve optimal printing [17,27,57].

### 2.2. Flexographic Printing

**Figure 3:**
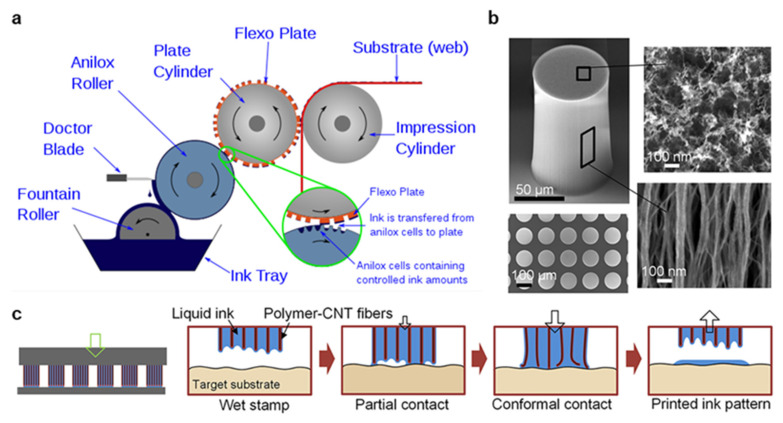
Advanced flexographic printing techniques. (**a**) Diagram of a standard flexographic printer to illustrate the key operating principles. (reproduced under creative commons license CC BY-SA 4.0). (**b**) Scanning electron microscope images of a CNT array (100 μm pillar diameter, 150 μm height) used for high-resolution flexography, and close-up top and side surfaces of a micropillar (reproduced with permission from *Langmuir* (2019), 35, 24, 7659–7671. Copyright 2019, ACS). (**c**) Simplified schematic of ink transfer from carbon nanotubes (CNTs) micropillar stamp loaded with ink. (reproduced with permission from *Langmuir* (2019), 35, 24, 7659–7671. Copyright 2019, ACS).

Flexography is another high throughput, a roll-to-roll fabrication method for printed electronics the origins of which can be traced to late 19th-century image printing [29]. Flexographic printing consists of five subprocesses, as depicted in Figure 3a. First, ink is pulled from a reservoir by the fountain roller. Second, the ink is transferred to an intermediate anilox roller containing millions of miniature engraved cells. Third, a blade removes excess ink from the anilox roller. Fourth, ink is transferred from the anilox to a flexible photopolymer plate containing a mirror engraved pattern. Finally, the substrate is rolled between the flexographic plate and an impression cylinder, yielding an ink deposition on the substrate. Because the printing roller is made of a flexible polymer wrapped around a metal cylinder, the prototyping and startup costs are significantly lower in flexography than gravure printing. However, plate deformation is a significant limitation to be overcome in high-resolution flexographic printing [41]. Another key difference between gravure printing and flexography is the presence of an anilox roll, which allows for a wider range of ink rheology to be printed, but whose geometry, pressure, and speed must be carefully optimized [41]. These challenges exist in addition to those faced by gravure printing, which is one explanation for the greater adoption of gravure for printed electronics. However, recent works have significantly improved flexographic printing capabilities, making flexography an exciting and fast-developing approach with a significantly lower barrier to entry than gravure printing. For instance, the surface energy of the flexography roll relative to the anilox and substrate can be modified to improve ink transfer in each phase, the print speed and pressure can be optimized for the specific transfer chemistry and pattern geometries, and the geometries themselves can be improved [41,58,59]. However, these innovations still result in resolutions >50 µm because of fundamental material limitations in the photopolymer flexographic roll. As a result, Kim et al. developed a microstructured, nanoporous carbon nanotube (CNT) stamp to replace the traditional roll with carefully controlled porosity, mechanics, and surface chemistry [60]. As shown in Figure 3b, the CNT nanopillars leave a precise open area in which the ink can reside. During printing, the stamp is brought into conformal contact with the substrate due to the mechanical flexibility of the CNTs, and a highly controlled deposition is produced as the stamp is removed, as shown in Figure 3c. This mechanism overcomes many of the key challenges in flexographic transfer by storing the ink in the stamp pores, then transferring directly to the substrate, and yield high-quality prints with a variety of nanomaterial inks of <20 µm were demonstrated [60,61]. In light of these innovations, flexography is now considered an exciting new field in printed electronics with great opportunities for further improvement.

### 2.3. Screen Printing

**Figure 4:**
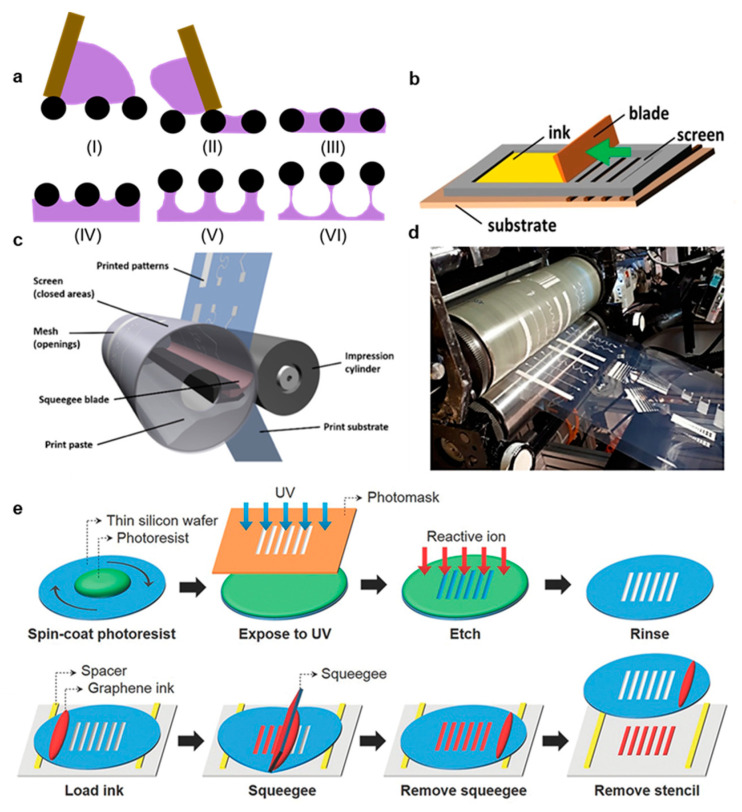
High-throughput screen-printing approaches. (**a**) Illustration of the six stages of screen printing, as proposed by Messerschmitt et al. and investigated Abbott et al. (**i**) Ink flooded into the mesh. (**ii**) Squeegee pressure brings the mesh in contact with the substrate. (**iii**) Ink adheres to both the substrate and mesh. (**iv**–**vi**) As the mesh is raised off the substrate, the ink first (**iv**) forms a continuous structure, then (**v**) forms filaments, which then (**vi**) collapse and level to form a deposition. (reprinted with permission from *ACS Omega* (2021), 6, 14, 9344–9351. Copyright 2021, ACS). (**b**) Illustration of a sheet-to-sheet screen-printer. (reprinted with permission from *Adv. Mater.* (2019), 31, 1806702. Copyright 2020, Wiley). (**c**) Illustration of a roll-to-roll screen printer, demonstrating the key operating principles. (reprinted with permission from Ind. *Eng. Chem. Res.* (2019), 58, 43, 19909–19916, Copyright 2020, ACS). (**d**) Image of a roll-to-roll screen-printer used in nanomaterial printing. (reprinted with permission from *Ind. Eng. Chem. Res.* (2019), 58, 43, 19909–19916, Copyright 2020, ACS). (**e**) Fabrication of a thin silicon screen printing stencil for high-resolution printing and printing process implanting this stencil. (reprinted with permission from *Adv. Mater.* (2014), 27: 109–115. Copyright 2014, Wiley).

Screen printing is an ancient printing method that has been employed in garment processing for centuries [19]. Today, it is a mature industrial process used in textiles, graphics, printed circuit silkscreens, in-mold electronics, capacitive touch sensors, printed heaters, and chemical sensors [47]. Significantly, screen printing is highly suitable for roll-to-roll manufacturing and high throughput processing. Unlike gravure printing and flexography, Screen printing involves the active transfer of ink from a mesh to a target substrate mediated by pressure and shear applied by a blade termed the squeegee [62,63]. Printing occurs in six distinct phases, as shown in Figure 4a [43,62]. First (I), ink enters the mesh after the application of gentle pressure such that it occupies the enter open mesh area, but does not run out from the bottom of the mesh [63]. Second (II), the mesh is brought into contact with the substrate as a result of applied pressure and the highly pseudoplastic ink becomes highly thin with applied pressure [64]. Third (III), the ink adheres to both the mesh and the substrate [43]. Fourth (IV), the mesh is pulled upwards as the squeegee progresses down the print, causing the ink to rise [43]. Fifth (V), the ink begins to form filaments underneath the mesh wires as the mesh is continually raised [43]. Finally (VI), the filaments break and the print levels, resulting in a deposition thickness that depends on the mesh open area and the ink adhesion to both the mesh and the substrate [43]. In traditional screen-printing applications, the substrate is placed on a flat plate below the mesh, as shown in Figure 4b [34]. In roll-to-roll screen-printing, the mesh is folded into a cylinder with the squeegee blade inside the cylinder [35]. The substrate is then rolled against the mesh and the impression cylinder, which causes an applied pressure against the squeegee and shear proportional to the print velocity. This process is depicted in Figure 4c, and an example roll-to-roll machine is shown in Figure 4d [35]. The same six steps described previously also apply to roll-to-roll screen printing, but some non-idealities in the mesh liftoff are caused by the curved substrate, especially when the radius of curvature is small [35,65].

In screen printing, the mesh height from the substrate and mesh geometry are crucial parameters, but the squeegee speed and pressure are not highly correlated with print quality [66]. This is because the sheer and compressive forces applied are typically large enough to elicit a strong sheer thinking response in ink and prevent ink hydroplaning before the squeegee [24]. Instead, optimizing ink rheology for this complicated fluid dynamics is crucial in screen printing [64]. Ink viscosities are typically high (10–30 PaS) and highly pseudoplastic so that they can avoid running through the mesh preprinting, flow easily during applied shear, and rapidly coalesce post print into a steep deposition without slumping on the substrate [11,67]. Furthermore, the mesh area that is not to be printed is blocked by an ultraviolet (UV) cured emulsion mask, and limiting this emulsion’s roughness is important in creating a high resolution and even print [66]. Like gravure and flexographic printing, the minimal resolution achievable in screen printing is limited fundamentally by the mesh quality, even if many inks with suboptimal rheology cannot approach this limit [63]. Specifically, screen printing meshes are limited by lithography resolution in emulsion etching, emulsion smoothness, and mesh geometries [11]. Creating a finer mesh with more weaves per unit area improves print resolution, but the reduction in mesh open area leads to a thinner print deposition [11]. Mesh counts generally reach their minimization limits beyond 140 threads per centimeter, and screens with around these mesh counts and optimized emulsions are capable of printing resolutions of around 70 µm [62]. However, Hyun et al. recently demonstrated a screen-printing stencil derived from a thin silicon wafer (90 µm thickness) with photolithographically defined openings to produce high quality depositions of graphene and AgNP inks with widths of 40 µm, and the silicon stencil fabrication and graphene printing is illustrated in Figure 4e [11]. In summary, screen printing is attractive for high throughput printed bioelectronics because it is a mature industrial process with significantly lower startup and prototyping costs than gravure and flexographic printing, and new innovations in mesh or stencil design open new opportunities for increased print resolutions.

### 2.4. Roll to Roll Inkjet Printing

**Figure 5:**
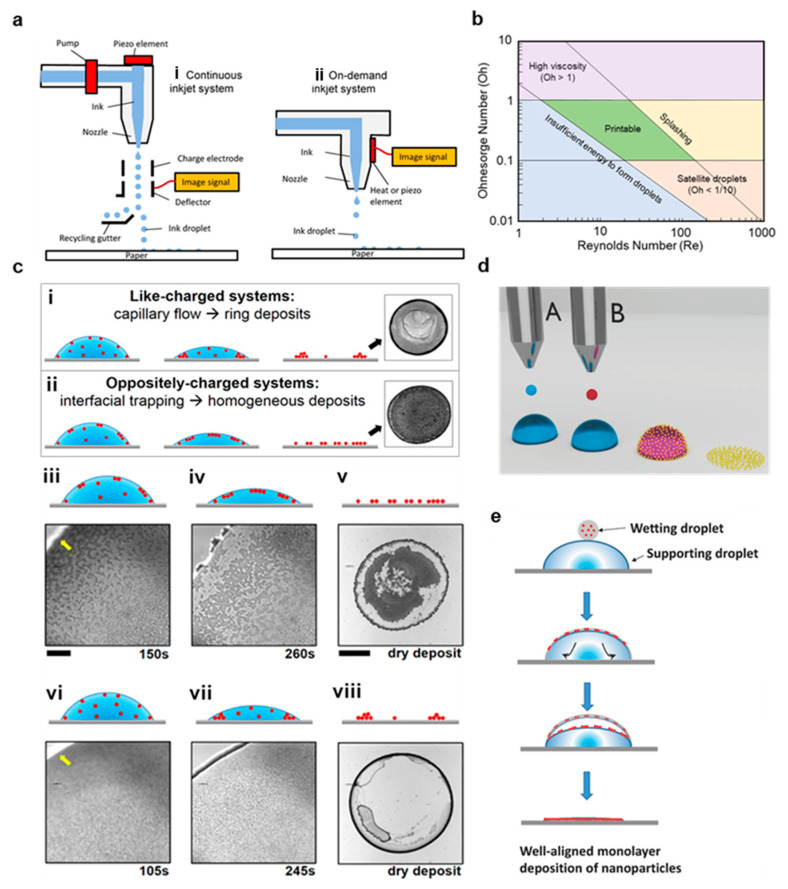
Roll-to-roll inkjet printing for hybrid bioelectronics. (**a**) Illustration of CIJ (**left**) and DOD (**right**) inkjet printing techniques. (reprinted with permission from *Micromachines* (2017), 8(6), 194. Copyright 2017, MDPI). (**b**) Reynolds number and Ohnesorge numbers that yield a high-quality inkjet deposition. Weber numbers can be calculated based on the ratio $Oh=\sqrt{We}$/Re. (**c**) Example images and illustrations of coffee ring formation due to capillary flow in evaporating droplets (**i**,**vi**–**viii**). This is compared to uniform depositions produced with added DTAB to promote particle trapping at the liquid–gas interface, which created particle skins that lead to homogenous disk like patterns upon drying (**ii**–**v**). (reprinted with permission from *Langmuir* (2015), 31, 14, 4113–4120, Copyright 2015, ACS). (**d**) Illustration of the dual drop inkjet printing process, where the blue ink is the supporting droplet, the red ink is the wetting droplet, and the gold represents the nanoparticles to be deposited. (reprinted with permission from *Adv. Mater. Interfaces* (2018), 5, 1701561. Copyright 2018, Wiley). (**e**) Illustration of the dual drop process used to deposit a uniform nanoparticle monolayer. (reprinted with permission from *Adv. Mater. Interfaces* (2018), 5, 1701561. Copyright 2018, Wiley).

Inkjet printing is an extensively developed technology that is widely employed in conventional printing applications, and it is exceptionally well suited to rapid, low-cost prototyping [25,31,68]. In inkjet printing, pressurized ink is forced through a nozzle, forming droplets that fall onto the substrate and collapse due to their momentum and substrate wettability [46]. Inkjet printing is achieved through two approaches, although drop on demand (DOD) printing is greatly preferred over continuous inkjet printing (CIJ) for bioelectronics because it allows for higher placement accuracy and higher resolutions [17]. In DOD printing, the ink is forced through the nozzle through either a contractile force applied from a piezoelectric actuator or a thermal disturbance that produces a shockwave capable of ejecting the ink [25]. In contrast, a CIJ printer charges ink droplets and continually passes them through an electric field formed between two deflection plates, allowing one to control the ink depositions [25]. Both inkjet printing processes are illustrated in Figure 5a. DOD printing is highly attractive because it allows for excellent control over deposition thickness, high resolution down to 40 µm, very inexpensive prototyping, and minimal startup costs, but it is also limited by clogging in the minuscule nozzle head, the uneven flow of material to the edge of the print in a process termed the coffee ring effect, and lower throughputs than the previously described methods [11,22,68]. 

Each of these limitations has been thoroughly studied using traditional graphics inks, and the challenge in bioelectronics fabrication is to apply these lessons to nanomaterial-based inks [25,69,70]. The fluid mechanics during printing is characterized primarily by three dimensionless quantities, the Weber number (We), Reynolds number (Re), and Ohnesorge number (Oh):

We=ζρv2γRe=ζρvηOh=WeRe=ηζργ

where *η*, *ρ*, and *γ* are the ink viscosity, density, and surface tension, respectively, *v* is the print velocity, and *ζ* is characteristic printing length, which is in most cases simply the diameter of the print head nozzle [25,26,70]. In almost all inkjet applications, Oh must be between 1 and 1/10 to achieve a quality print, as illustrated in Figure 5b [28]. At high Oh values, the ink viscosity will prevent stable drop formation [28]. When Oh is too low, the ink forms many uncontrolled drops instead of a single, well-defined drop, which results in an unusable print [28,69]. In addition, the particle size cannot be >*ζ*/50 in order to avoid immediate nozzle clogging [71]. As we will discuss in Section 3, these requirements greatly complicate the printing of Ag nanowires (AgNWs) and CNTs, which are usually much longer than *ζ*/50, and carbon-based nanomaterials, which are difficult to disperse with both low viscosity and high material loadings [47,72,73]. Another crucial challenge in inkjet printing is the accumulation of the deposited material along the edge of the print, commonly termed the coffee ring effect [48]. This occurs when the edge of a droplet on a substrate is fixed in place and capillary flow induced by evaporation of the drop causes material to flow from the interior towards this fixed edge [48]. This process is combatted by Marangoni flow within the drop, but many surfactants and even added water tend to have very weak Marangoni flows [74]. There are numerous methods employed to combat coffee ring formation, including careful control of the surfactant mediated interactions between particles and the liquid–gas interface [72,74], mixing high and low boiling point solvents [75], heating the substrate [76], depinning the contact line (which reduces print definition) [77], alternating voltage electrowetting [78], and dual drop inkjet printing [33]. In an example of the first method, Anyfantakis et al. mixed surfactants and colloids with opposite charges and observed that particles that absorbed the surfactants become hydrophobic, giving them a greater affinity to the liquid–gas interface [72]. These particles on the drop surface prevented capillary flow from collapsing the structure, leading to a uniform deposition, as shown in Figure 5c [72]. In the later method, two main approaches are employed. First, the Langmuir–Blodgett concept is applied to the picolitre depositions by first depositing a supporting layer, then adding a functional ink on top containing colloidal nanoparticles that assemble as the solvent dissolves to produce a highly uniform layer, as illustrated in Figure 5d,e [33]. Second, antisolvent crystallization can be used to form highly uniform semiconducting films at the liquid–air interface in a mixed droplet [79]. This occurs after printing an antisolvent layer, then a semiconductor solution. The undissolved nuclei form a cohesive film on the drop surface, preventing the drop from collapsing as the solvent evaporates [79].

Finally, significant commercial interest in inkjet printing has led to many efforts to improve manufacturing throughput, and numerous inkjet printers can achieve speeds far beyond those achieved in home-use graphics printers [73]. However, there are still key tradeoffs between print resolution, deposition uniformity, and throughput [26,73]. The greatest improvements in throughput generally come through roll-to-roll processing, stringent quality control on component manufacturing, and precise temperature control, all of which have been thoroughly investigated by private companies [73]. Even in the most advanced systems, nozzle clogging is still a crucial issue with nanomaterial inks that limits manufacturing throughput, and continuous cleaning of the systems is therefore necessary [73]. Inkjet printing is highly attractive for printing bioelectronics because complex systems can be very rapidly prototyped during development, then easily scaled to mass production, but there are also very strict requirements on nanomaterial ink properties, lower demonstrated throughputs than alternative methods, and key challenges relating to nozzle clogging that complicate high throughput fabrication.

### 2.5. Slot Die and Blade Coating

Slot die and blade coating, which are sometimes referred to as bar coating or knife coating, are high throughput methods to deposit homogenous films for applications that do not require complex patterns to be formed [34,49]. In blade coating, ink is placed before the blade, and deposition is left as the blade swipes across the substrate [34]. The thickness of the resultant deposition depends largely on the blade height relative to the substrate, the print velocity, ink viscosity, and ink-substrate wetting contact angle [34]. In slot coating, ink is continually pumped from a slot inside a print head, which can be masked to print unidirectional lines [49]. In addition, the print head can be displaced perpendicular to the print direction to yield curved lines [80]. The print quality and film thickness in slot die coating is determined by the meniscus forming between the print head and the substrate, and this meniscus can be controlled by the same parameters mentioned for the doctor blade in addition to the pumping rate and temperature control of the ink [30]. Slot die and blade coating are mature manufacturing processes for depositing homogeneous films, which are desired in pressure, chemical, and electrophysiological sensors for soft bioelectronics; however, these methods are not well suited to more complicated printing applications that require sophisticated patterning.

## 3. Conductive Nanomaterial Printing

### 3.1. Fundamentals

Printed nanomaterial applications typically follow the same four-step process: first, nanomaterials are produced either through top-down methods, where the nanomaterial is broken off from bulk material, or bottom-down approaches, where the particles are synthesized from atomic precursors [81,82,83,84,85]. Second, these nanomaterials are dispersed in printable inks with viscosities and rheology that are optimized for the printing method of choice [11,26,86]. Third, the inks are printed on a substrate and create a deposition based on the fluid mechanics during printing and free energy effects at the liquid–gas and liquid–solid interfaces [11,44,63,69]. Finally, the solvent is evaporated, and, in some cases, the nanomaterials are sintered to yield a conductive structure [50,87,88]. When choosing a nanomaterial for a specific bioelectronics’ application, the material’s electrical and mechanical properties, the tendency to agglomerate, required loading to produce a conductive network, and particle aspect ratio are crucial considerations. For instance, graphene nanoplatelets and carbon nanotubes (CNTs) have excellent conductivities, are easily functionalized, and have high durability, but they are difficult to disperse in printable inks because of strong intermolecular forces [67,83,89]. In most nanomaterial inks, the solvent is highly polar, and the nanomaterial is nonpolar [90,91]. An amphiphilic dispersion agent, such as polyvinyl pyrrolidone (PVP) and sodium dodecyl sulfate (SDS), is introduced [64]. The nonpolar region binds to the nanomaterial surface, leaving a polar tail that allows the material complex to be dissolved in the solvent and creates interparticle repulsive forces that prevent agglomeration. In silver nanomaterials, PVP is highly attractive because the nitrogen and oxygen atoms enable effective absorption into the surfaces of Ag seeds or particles, whereas SDS is effectively absorbed into CNT surfaces in the presence of ultrasonication energy [64,91,92]. However, SDS is not biocompatible, and it must be effectively removed or reduced in concentration either before or after printing if the CNTs will be skin-contacting [91]. On the other hand, PVP is biocompatible, making it more attractive for many bioelectronics applications [93]. In the following sections, we will summarize recent developments in the synthesis, dispersion, high throughput printing, and sintering for each nanomaterial and demonstrated the soft electronics devices created with these methods.

### 3.2. Metal Nanoparticles (NPs)

#### 3.2.1. Material Properties, Synthesis, and Ink Formation

**Figure 6:**
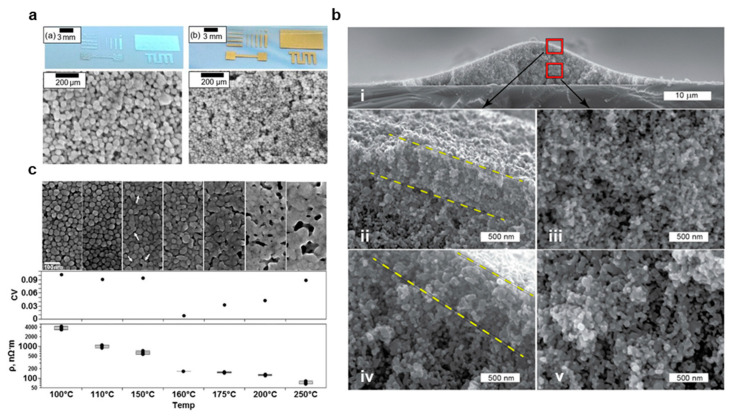
Metal nanoparticles for printed electronics. (**a**) photos and SEM images of inkjet-printed films with Ag (left) and Au (right) NPs. (reprinted under Creative Commons license CC BY 4.0 from *Adv. Radio Sci.* (2019), 17:119–127.) (**b**) SEM images of an AgNP deposition cross-section, showing the overall structure (**i**), surface (**ii** and **iv**) and interior (**iii** and **v**) after rapid laser sintering. (reprinted with permission from *Appl. Sci.* (2020), 10(1), 246. Copyright 2019, MDPI). (**c**) SEM images of AgNP films sintered at various temperatures, with corresponding graphs depicting the coefficient of variance and resistivity (reprinted with permission from *Materials* (2011), 4(6), 963–979, Copyright 2011, MDPI).

AgNPs and CuNPs are low aspect ratio particles, typically with spherical geometries and radii from 10–100 nm for printing applications, that are often formed through wet chemistry from ionic precursors [85]. Example images of printed Ag and Au nanoparticles with spherical geometries are shown in Figure 6a [94]. In wet chemistry NP synthesis, a metal ion precursor, such as AgNO_3_ and Cu(NO_3_)_2_, is reacted with a reducing agent, such as ethylene glycol (EG) or ascorbic acid in solution with a capping agent, such as PVP and SDS [85]. In addition to wet chemical synthesis, NPs may also be formed through physical methods, such as evaporation condensation [95] and laser ablation [96], additional chemical methods, such as microemulsion [97], UV or other photonic source initiated photoreduction [98], electrochemical synthesis [84], irradiation [99], microwave-assisted synthesis [100], and biosynthesis techniques, either through bacteria, fungi, algae or plants [100]. Spherical metal NPs tend to agglomerate strongly because of their large surface areas, strong interparticle attractions, and particle symmetry regardless of orientation. As a result, the NP surface must be functionally modified to aid in dispersion. Furthermore, their low aspect ratios require high material loadings in order to form conductive networks, but loadings over 60% complicate the design of inks for printing methods requiring low viscosities or which tend to clog, such as inkjet printing. On the other hand, the excellent material symmetry, high material loading, and surface pre-melting allow for very effective, low-temperature sintering at around 200 °C into uniform conductive films. An example of a printed AgNP film is provided in Figure 6b, and the resultant AgNP network after the solvent is dissolved is readily seen [101].

NPs inks are typically synthesized with 40–88% material loadings and dispersed with high concentrations of dispersants, such as 1:1 PVP mixtures. Because this drastically reduces the ink viscosity, PVP concentrations must be limited for screen printing. For instance, Wang et al. preheated and magnetically stirred a 0.3 M solution of PVP and ethylene glycol (EG) to increase the ability of PVP to bind to the AgNP surface, allowing them to disperse the NPs with a 1:2 PVP/AgNO_3_ ratio [102]. After mixing 60 mL of 0.3 M PVP-EG solution and 40 mL 0.29 M AgNO_3_-EG, the solution was mixed with N,N-dimethylformamide, hydroxyethyl cellulose, and ethylene glycol (EG) to yield a 45 wt.% ink with viscosity and rheology optimized for screen printing. When printed on PI, and sintered at 220 °C, the inks demonstrated a remarkably low resistivity of 8.3 × 10^−6^ Ω∙cm, which is only five times greater than the bulk silver resistivity [102]. For gravure printing, Shiokawa et al. created an organic protection layer on AgNPs to improve dispersibility and printability. AgNO_3_ (22 wt.%) was mixed with oxalic acid dihydrate (9 wt.%), n-Hextlamine, N,N-dimethyl-1,3diaminopropane and oleic acid, and AgNPs were synthesized through thermal decomposition of an oxalate-bridged silver alkylamine complex [103]. The resultant powder was then dispersed in tetralin, tetradecane, and dodecane with 80 wt.%, and it was determined that the tetralin solution had the highest printability [103]. The ink was then gravure printed on a glass slide with widths of 20 µm with 4.4 µΩ cm [103]. For flexographic printing, Benson et al. developed an AuNP ink that was used to create biocompatible sites on a PI substrate for the enzyme attachment in glucose sensing [104]. AuNPs were synthesized by reducing HAuCL_4_ (0.2 g) with NaBH_4_ (0.05 g) in the presence of PVP (0.15 g) in 30 mL DI. The solution was centrifuged to yield an AuNP pellet, which was subsequently redispersed in 70% IPA and 30% dionized water (DI) via ultrasonication. Electrodes were fabricated by flexographic printing of a carbon layer, then the AuNP layer, with a printing force of 125 N, anilox force of 125 N, and speed of 0.6 m/s. After functionalization with glucose oxidase, the electrodes demonstrated a high sensitivity of 5.52 μA mM^−1^ cm^−2^ with a detection limit of 26 μM [104]. In addition, NPs are highly attractive for inkjet printing because of their low aspect ratios, which can avoid nozzle clogging, and they have thus been carefully studied [26]. For instance, Fernandes et al. designed an experiment to assess the printability and conductivity of AgNP inks with a variety of solvents and additives [47]. Silver nanoparticles were synthesized by reduction of 100 mL 0.006 M AgNO_3_ and 0.008 M PVP in DI water by 8 mL of 0.529 M sodium borohydride (NaBH_4_), centrifuged at 1500 RPM for 1 h, then dispersed in a range of ethanal based solutions with viscosities ranging from 3.7–7.4 mPa.s and material loadings from 8–16 wt.% [47]. It was determined that the EG, ethanol, ethanolamine, and hyperdispersant (Solsperse 20000) ink with 5.25 mPa.s viscosity resulted in the greatest printability due to the addition of humectants (i.e., ethylene glycol and ethanolamine) combined with low resistivity (1.6 × 10^−4^ Ω.cm) [47] Finally, AgNPs are not well suited to skin contact because of poor biocompatibility, so they either must be well insulated or replaced with AuNPs for such applications [26].

#### 3.2.2. Post Print Processing

After printing, NP depositions must be cured to remove the solvent, and many, but not all, inks are also sintered to form conductive sheets [94,100]. Sintering is not typically employed for printing on TPU, PET and paper because of low substrate melting points, in printing stretchable interconnects, where the unconnected particles form effective conductive networks with strain, and for biosensor applications where increased surface area is preferred (e.g., glucose sensors) [104]. Sintering, however, is highly advantageous for forming conductive sheets with low resistances and high yield stress [47,105,106]. The SEM images in Figure 6c clearly show the formation of a more uniform metal sheet with increased temperature in thermal sintering, and this is reflected in the decreased resistivity [107]. Although thermal and chemical sintering are easily employed in sheet-to-sheet processes, alternative methods are needed for roll-to-roll integration [105]. One approach with significant promise is photonic sintering, where energy is provided by an ultrafast pulsed laser source with a wavelength tuned to match the ink’s absorption spectrum [105,106]. For instance, Hösel et al. demonstrated a single exposure system integrated into roll-to-roll flexography printing with speeds of 2.5 m/min [106]. In addition, electric, plasma, and microwave sintering are well suited for roll-to-roll processes [108,109]. Allen et al. demonstrated effective electric sintering with a directly applied voltage, but the method has not been explored for roll-to-roll processes, likely because of the need to create direct and secure contact between the pattern and electrode [108]. In contrast, indirect methods, such as microwave sintering, can be well integrated into roll-to-roll processes, but their throughput is greatly limited compared to photonic and electrical methods [105]. For instance, Fujii et al. demonstrated effective sintering in 1.5 min, compared to milliseconds in other methods [110]. Finally, plasma sintering is a promising sintering method, but it is limited for thick or multilayer depositions by a slow depth penetration, which is an issue for high throughput applications [105].

### 3.3. Metal Nanowires (NWs)

#### 3.3.1. Material Properties, Synthesis, and Ink Formation

**Figure 7:**
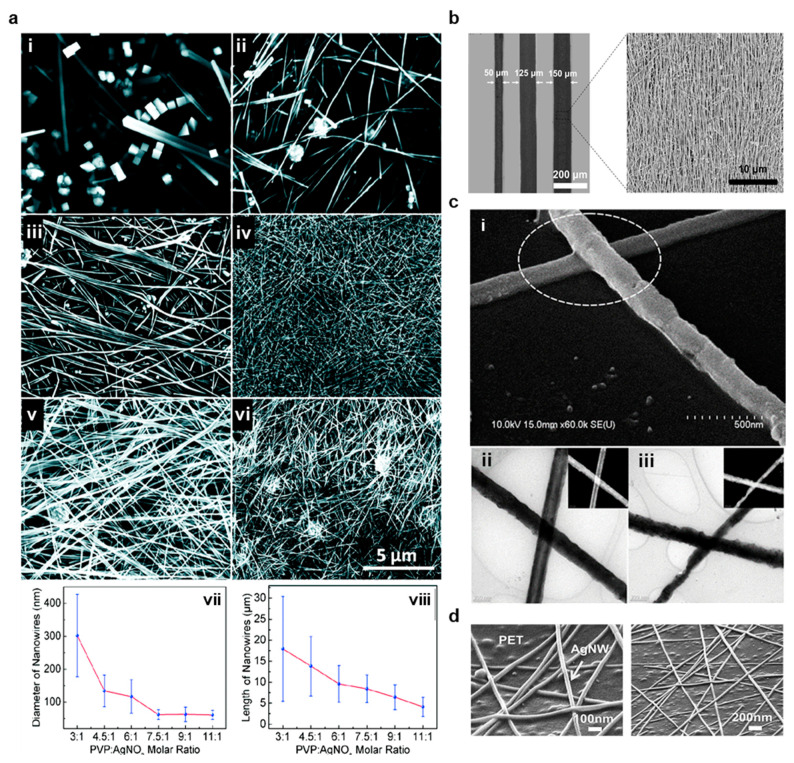
Metal nanowire synthesis, printing, and welding. (**a**) SEM images of Ag nanowires synthesized at different PVP:AgNO3 molar ratios of (**i**) 3:1, (**ii**) 4.5:1, (**iii**) 6:1, (**iv**) 7.5:1, (**v**) 9:1, and (**vi**) 11:1. All scales are the same. Changes in (**vii**) nanowire diameter and (**viii**) length with PVP:AgNO3 molar ratio are also shown. (reprinted with permission from *Cryst. Growth Des*. (**2011**), 11, 11, 4963–4969, Copyright 2011, ACS). (**b**) Images of gravure printed AgNW traces with various thicknesses and an SEM image demonstrating the aligned AgNW network. (reprinted with permission from *Sci. Rep*. (2018), 8, 15167, Copyright 2018, Nature Publishing Group). (**c**) SEM image of CuNWs nanowelded with laser irradiation (**i**) and TEM images of CuNWs before (**ii**) and after (**iii**) laser irradiation. (reprinted under Creative Commons license CC BY from *Sci. Rep.* (2017), 7(1)). (**d**) SEM images of sparse, randomly oriented AgNWs on a PET film (reprinted courtesy of *Org. Electron*. (2014) 15(11), 2685–2695, Copyright 2014, Elsevier).

Unlike NPs, NWs are differentiated by their large aspect ratios, with lengths often 1000 times greater than their widths [3,11,111]. As a result of these aspect ratios, NWs can from conductive networks with very minimal loading, exhibit minimal bending stiffness and exceptional yield strength approaching the theoretical value of E (Young’s modulus)/10, high optical transmittance, and electrical conductivities that are dominated by quantum effects [17,45,112]. When the NW widths become too small, conductivity is greatly diminished by edge effects from atoms at the material surface and scattering, setting a practical limit on widths for printed inks [112]. Compared to NPs, NW inks are significantly easier to synthesize because NWs in random orientations are much more resistant to agglomeration [11]. Unlike sintered NP sheets, these NW networks can stretch and deform when embedded in a polymer matrix [45]. NWs can also be made biocompatible because of the inability of small Ag particles to migrate into the skin [111]. In addition, NWs may be laser welded for the rapid formation of highly conductive sheets. Nanowires are traditionally synthesized through the polyol method for printed inks, but the template method is also widely employed [11,17,113]. In polyol synthesis, the solution temperature, PVP molar ratio to AgNO_3_, stirring rate, the introduction of platinum seeds or other nucleation agents, and the addition of chloride or bromide ions can all be used to control the material dimensions and AgNW quality [93,113,114]. Figure 7a shows SEM images of AgNWs synthesized in various PVP solutions along with quantitative measurements of average NW diameter and length, and this experiment demonstrates that PVP solutions must be carefully optimized for Polyol synthesis [93]. During this synthesis method, AgNWs are typically produced from Ag seeds reduced from AgNO_3_, and these seeds are capped by the presence of PVP [93,114]. Although the exact mechanism by which AgNWs are synthesized in the polyol process is not fully known, it is likely that the differential affinity of PVP to the <100> plane than <111> plane in silver leads to unidirectional growth [115]. Despite a rigorous theoretical model, empirical findings allow for precise control of material aspect ratios and purities [93].

AgNW inks typically contain much lower material loadings than AgNP inks, simplifying ink design. As a result, greater resolutions are often achievable. For instance, Liang et al. experimented with different material loadings in AgNW inks for high-resolution screen printing [11]. AgNWs with aspect ratios of 500 were mixed with (hydroxypropyl)methyl cellulose (HMC), Zonyl FC-300, and defoamer MO-2170 in a distilled water solution and sonicated [11]. HMC is a viscoelastic polymer with hydroxy groups that bind strongly to AgNWs to aid in dispersion and that serves as an emulsifier and thickening agent [11]. Zonyl FC-300 was used to decrease the surface tension of the ink and promote substrate wettability for high-resolution printing, and defoamer MO-2170 was necessary to prevent foaming during mechanical agitation. It was determined that a 6.6 wt.% AgNW ink had the greatest pseudo-plasticity and lowest viscoelasticity (i.e., the ink had the highest difference in viscosity during low and high shear and recovered viscosity the quickest after applied shear was removed), which allowed for screen printing of highly conductive (4.67 × 10^4^ S/cm) 50 µm width traces [11]. Likewise, Huang et al. investigated various material loadings for gravure printing AgNW inks, arriving at an optimal value of 5.0 wt.% that yielded 50 µm width traces and 5.34 × 10^4^ S/cm conductivity [50]. AgNWs were synthesized in the presence of PVP (50 mL 0.09 M in EG) and NaCl (150 µL 0.1 M in EG) and centrifuged with acetone and ethanol to remove the solvent and surfactant. The AgNWs were then dispersed in a Poly(ethylene oxide) solution. At 1.5 mm/s, the 5.0 wt.% ink demonstrated a capillary number of 1.09 and viscosity of 20.9 Pa.s, making it suitable for gravure printing [50]. Optical (left) and SEM (right) of the resultant prints are shown in Figure 7b [50]. It is also possible to pattern a nanowire precursor on a substrate and grow the nanowires in situ, and this process has been demonstrated using flexography for ZnO NW functionalization of electrochemical biosensors [116]. In this work, commercially available carbon and AgCl inks (Gwent, PontyPool, UK) were flexographically printed on flexible PI to form a conductive electrode, and the ZnO precursor ink (1.1 g of zinc acetate in 10 mL DI and 40 mL IPA) was printed with an anilox volume of 12 cm^3^/m^2^, anilox force of 125 N, printing force of 150 N and printing speed of 0.2 m/s. The ZnO wires were hydrothermally synthesized in situ in an aqueous solution of 10 mM hexamethylenetetramine to yield a flexible glucose sensor with a sensitivity of 1.2 ± 0.2 µA mM^−1^ cm^−2^ with a linear response to the addition of glucose over a concentration range of 0.1 mM to 3.6 mM [116]. Finally, inkjet printing AgNWs have been demonstrated, but the printing process must be carefully controlled to prevent nozzle clogging. In a sheet-to-sheet process, Al-Milaji et al. created an AgNW ink for inkjet printing by synthesizing AgNWs with an average of diameter of 100 nm and length of 14.5 µm in a polyol process, then dispersing the resultant precipitate in ethanol [45]. The resultant ink was printed on an uncured liquid PDMS layer spin coated on PET, and the AgNW ink was absorbed into the PDMS to create a stretchable interconnect. The connectors demonstrated high reliability during strain and bending, but initial resistances were high (0.68 kΩ over 25 mm) [45]. In contrast, Finn et al. sonicated commercially purchased NWs to reduce particle length, dispersed in IPA, and optimized inkjet parameters to yield sheet resistances of 8 Ω/sq and conductivities of 105 S/m in traces with widths of 1–10 mm and thickness of 0.5–2 µm after curing at 110 °C [117]. In order to reduce clogging, a Dimatix printer with 16 nozzles of diameter 21.5 µm spaced 254 µm apart was used to create 10-pL droplets at 5 kHz with a spacing of 20 µm and 50% overlap [117].

#### 3.3.2. Post Print Processing

NW networks are often cured without sintering because the particles naturally contact when randomly dispersed, but welding NWs can significantly reduce wire-to-wire resistances when significant PVP coatings are present. For instance, Lee et al. used thermal sintering at 200 °C for 20 min to reduce resistance in a printed AgNW trace from 1000 to 100 Ω/sq [118] and Li et al. photonically welded NWs to reduce sheet resistances from 53 to 7.1 Ω/sq [23]. An example SEM image of laser-welded AgNWs is provided in Figure 7c [119]. Finally, NWs can be welded by NPs embedded in a matrix film. In one demonstration, Triambulo created a highly conductive (5.0–7.3 × 10^5^ S/m) AgNW-AgNP matrix film on a flexible PET substrate with similar optical transmittance (>90%) compared to a pure AgNW film, and SEM images of the resultant network are provided in Figure 7d [120]. Despite the advantages of welding NWs for improving conductivity, the ability to process NWs at room temperature for many ink formulations is a key advantage in roll-to-roll integration, especially when attempting to limit start-up costs [11].

### 3.4. Graphene

#### 3.4.1. Material Properties, Synthesis, and Ink Formation

**Figure 8:**
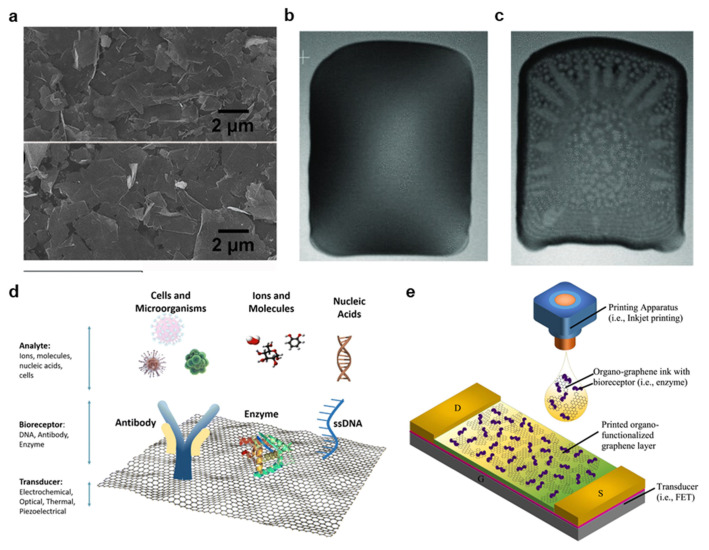
Graphene printing and functionalization for bioelectronics sensors. (**a**) Atomic force microscopy (AFM) images of screen-printed graphene (reprinted with permission from *J. Colloid Interface* Sci. (2021), 582(A), 15. Copyright 2021, Elsevier). (**b**,**c**) SEM images of an inkjet-printed graphene deposition (**b**) before and (**c**) after curing, with a minor coffee ring effect. (reprinted with permission from *Adv. Mater.* (2013), 25(29), 3985–3992. Copyright 2013, Wiley). (**d**) Examples of covalently bonded bioreceptors on a functionalized GO deposition. (reprinted with permission from *J. Nanobiotechnol.* (2018), 16,75. Copyright 2018, Springer Nature). (**e**) Graphical depiction of a FET biosensor with organo-functionalization. (reprinted with permission from *Biosens. Bioelectron.* (2017). 87, 7–17. Copyright 2017, Elsevier).

Graphene is a zero-gap semiconductor with exceptional conductivity, biocompatibility, and high mechanical strength that is easily functionalized with numerous materials for sensing applications [81]. As a result, graphene is highly attractive for printed bioelectronics [53,67,86]. However, graphene is very difficult to print because of its low dispersibility in printable inks [86]. Typically, graphene is formed through exfoliation from graphite, either through ultrasonic or mechanical methods, but numerous additional mechanisms have been explored, including electrochemical synthesis, chemical vapor deposition, laser processing and sodium ethoxide pyrolysis [81]. An example SEM image of graphene sheets for screen printing is provided in Figure 8a [121]. Once synthesized, dispersing graphene in printable inks is a key challenge. Although graphene oxide (GO) is easily dispersed, it must be reduced after printing, limiting throughput, and creating numerous defects that detract from the material’s electrical and sensing capabilities.

To create a screen printable ink, He et al. dispersed 5 g of graphene nanoplatelets (GNPs) in 50 mL EG and 0.5 g PVP and printed traces on the PI with conductivities of 8.81 × 10^4^ S/m [67]. Graphene’s natural tendency to agglomerate was used to increase the ink viscosity to a range reasonable for screen printing (1 Pa·s) [67]. For gravure printing, Secor et al. noted that stabilizing graphene with ethyl cellulose (EC) greatly aids in dispersion [53]. Graphene was exfoliated from graphite in ethanol with EC, excess graphite was removed by centrifuging. The resultant graphene–EC precipitate was then redispersed in ethanol and terpineol, and the specific quantities of each substance were altered to yield inks with various viscosities [53]. As described in Section 2.1, the optimized ink was able to be printed with trace widths of <30 µm with conductivities >10,000 S/m [53]. Although initial feasibility studies have investigated flexographic graphene printing, no successful use in soft bioelectronics has been reported to date [122]. Likewise, many inkjet applications select to use GO instead of graphene, but graphene printing has been successfully reported. For instance, Li et al. exfoliated graphene from graphite in dimethylformamide (DMF), and the toxic DMF is distilled out in a terpineol solution [86]. Graphene in this state is normally stable for only hours, but in this work, EC was added to protect the graphene from agglomeration. After the solvent exchange, the graphene/toluene dispersion was mixed in ethanol in a volume ratio of 3:1 to yield a printable viscosity and rheology. Figure 8b,c depicts this optimized ink (b) directly after printing and (c) after curing, demonstrating a mostly uniform deposition with a minimal coffee ring effect [86]. Finally, the resultant ink was printed in 80 µm traces on both plastic, and silicon substrates and printed supercapacitors were able to achieve a specific capacitance of 0.59 mF cm^−2^ [86].

#### 3.4.2. Laser Synthesis of Graphene

Laser printing is an emerging technology whereby a thin film of material is selectively removed from a carrier substrate via a laser beam and irradiated to a receiver substrate [123]. This approach allows for integration with roll-to-roll laser printers, printing without harsh chemicals, high spatial resolution, and control of edge plane functionalization, which makes laser printing of great interest for bioelectronics applications [123,124]. For instance, Rahimi et al. demonstrated a high throughput process by which graphene can be irradiated onto a PDMS substrate to yield strain sensors sensitive up to 100% strain with a gauge factor of up to 20,000 [124]. It was reported that laser power and speed greatly affected print quality and conductivity, and the authors optimized the process to 0.5–1.9 m/s and 4.5–8.25 W for printable traces [124]. Laser printed graphene is also of great utility in a number of biosensor applications. For instance, Ortiz-Gómez et al. ablated a PI film with a 12 W CO_2_ laser operating at 2.4 W and 0.15 m/s to create a graphene heater for a microfluidic device that used fluorescent silicon nanodots to detect total carbohydrates [125]. In addition, GO can be reduced by laser excitation through the conversion of sp^3^ carbon to sp^2^ and the removal of oxygen functional groups, and the photothermal and photochemical processes involved in the reduction of GO can be well controlled by altering the laser wavelength [126]. For instance, Zahed et al. used a CO_2_ laser to reduce GO for an electrocardiography (ECG) sensor with comparable signal quality to commercial Ag/AgCl electrodes (12.9 dB vs. 13.3 dB) [127].

#### 3.4.3. Post Print Processing

Because graphene ink stability is predicated heavily on the addition of strong solvents and polymer stabilizers, these chemicals must be evaporated, dissolved, decomposed, or otherwise removed in order to yield optimal conductivity and material properties [128]. Post-print processing is highly dependent on the choice of chemical additives. For instance, graphene dispersed in high concentrations of EC must be treated at 300–400 C, whereas EG-PVP mixtures can be cured at 120 °C [82]. Furthermore, several inks that do not evaporate solvents can be treated at room temperature, although these inks will exhibit lower conductivities as a result [129,130]. While thermal curing beyond 120 °C is not suited for flexible electronics on many substrates, such as PET and TPU, novel laser treatment approaches are able to efficiently treat printed patterns without damaging the underlying substrate [131]. For instance, Jabari et al. reported a laser treatment method to cure printed graphene with similar conductivities to traditional thermal curing [131]. Likewise, Secor et al. demonstrated an intense pulsed light annealing for inkjet-printed graphene that is suited to a variety of substrates and can result in fewer impurities than thermal alternatives [132]. Finally, GO is much easier to disperse than graphene, but it must be reduced after printing with harsh chemicals and high temperatures, which often results in defects and poor conductivity [128]. As a result, GO is not as attractive as graphene for bioelectronics applications.

#### 3.4.4. Graphene Functionalization for Biosensor Applications

Organo-functionalized graphene has played a crucial role in the development of novel biosensors, and the ability to print such sensors in roll-to-roll methods would have a transformative effect on healthcare [12]. Although each of the four materials covered in this review has been successfully explored biosensors, those based on graphene have generated the most recent interest because of the great degree of freedom in material functionalization [15]. Graphene functionalization occurs either covalently or non-covalently. In covalent functionalization, graphene is oxidized to GO, and covalent bonds are formed to organic functional groups on a sensing material [128]. For instance, a carboxylic group on GO can covalently bond to glucose oxidase to form a glucose sensor [12,16,128]. Examples of covalent sensing systems on functionalized GO are illustrated in Figure 8c [13]. Non-covalent bonding occurs when functional groups are attracted to graphene through Van der Waals and electrostatic forces, but this bonding is typically nonstable for long durations [12,133]. Instead, target biomolecules may be directly absorbed into the graphene, allowing the graphene to serve as a sensor through non-covalent functionalization [133]. The most common form of glucose-based biosensors is electrochemical sensors. Graphene functionalized with biological receptors is employed as a working electrode to detect analytes through electrochemical oxidation or reduction of analytes [12]. For instance, Kinnamon et al. screen-printed GO on a textile substrate and bound 1-Pyrenebutyric acid-*N*-hydrosuccinimide ester (PANHS) as a crosslinker to bind to an influenza A-specific antibody [16]. The textile sensor demonstrated high stability with washing (~4.6% variability) and accurate sensing over a range of virus expression of 10 ng/mL to 10 μg/mL with a limit of detection of 10 ng/mL. The sensor also exhibited very good specificity, and the sensing range is well suited to the average human viral expression of 50 ng/mL [16]. In addition, graphene field effect transistor (FET) biosensors may be used to control the flow of current as a function of charge accumulating on a functionalized graphene gate of channel, as shown in Figure 8d [12]. For instance, Xiang et al. used inkjet printing to deposit a graphene channel for a fully printed FET on the PI with low resistivity (110 Ω/sq) that was subsequently functionalized in cystamine solution (Figure 8e) [133]. Norovirus antibodies were then bonded, and bovine serum albumin was introduced to prevent non-specific binding of other biomolecules. It was determined that the voltage gain from source to drain with an applied 10 GHz wave generates a linear response from 0.07 to 3.70 dB when the concentration of Norovirus protein increases from 0.1 to 100 μg/mL [133].

### 3.5. Carbon Nanotubes

#### 3.5.1. Material Properties, Synthesis, and Ink Formation

**Figure 9:**
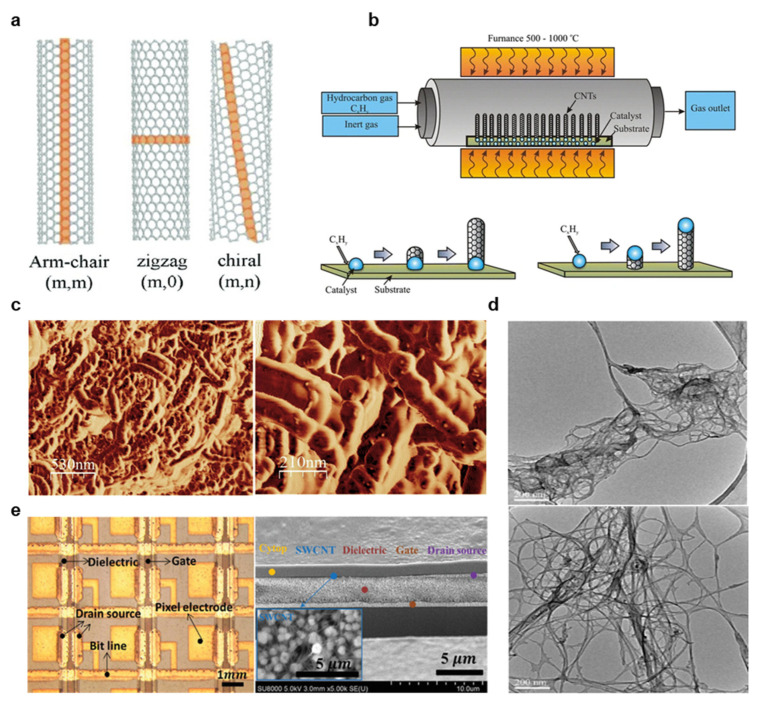
Carbon nanotubes for high throughput bioelectronics printing. (**a**) Armchair, zigzag, and chiral CNT geometries, each of which exhibits unique material properties. (reprinted with permission from *Physica E Low Dimens. Syst. Nanostruct*. (2014), 59:186–191. Copyright 2014, Elsevier) (**b**) Schematic representation of the CVD process for CNT synthesis, with illustrations of the base growth (bottom left) and tip growth (bottom right) CNT synthesis methods. (reprinted with permission from *Chem. Biol. Technol. Agric.* (2016), 3(17). Copyright 2016, Springer Nature) (**c**) AFM images of printed MWCNTs at different magnifications. (reprinted with permission from *RSC Adv.* (2017), 7, 44076–44081. Copyright 2017, RSC). (**d**) TEM images of SWCNTs in an SDS solution after sonication for 4 h (top) and 6 h (bottom). (reprinted with permission from *J. Surf. Eng. Mater. Adv. Technol.* (2013), 3, 6–12. (**e**) Image of pixels in a roll-to-roll gravure printed TFT-active matrix with 10 PPI resolution (left) and cross-sectional FIB-SEM of printed SWCNTs on the printed dielectric (right). (reprinted with permission from *Adv. Electron. Mater.* (2020), 6, 1901431, Copyright 2020, Wiley).

CNTs offer very attractive elasticity, biocompatibility, surface area, aspect ratios, strength, and conductivity, making them of great interest for electronics applications, but very strong van der Waals interactions greatly complicate particle dispersion [83]. CNTs consist of rolled graphene sheets that consist of either one tube (single-walled CNT, or SWCNT) or multiple tubes (multi-walled CNT, or MWCNT) held together with Van der Waals attractions [90]. The direction in which CNTs are rolled greatly affects their observed properties, and illustrations of several common orientations are provided in Figure 9a [134]. “Armchair” CNTs are highly preferred for interconnects or conductive planes because their identical chiral indices create highly uniform conductivity [92], but zigzag or chiral CNT orientations are widely employed for their semiconducting effects, and they are also of great interest for printed transistor fabrication [91]. CNTs are typically synthesized through three processes: chemical vapor deposition (CVD), arc discharge, and laser ablation, although CVD is the most widely employed. In CVD, metal NPs of the CNT diameter are introduced in the presence of a carbon-based gas, such as CO_2_, to form CNTs, and this process is illustrated in Figure 9b [82,91]. In order to remove the NPs and other impurities, the CNT powder is typically sonicated or treated with acid, and CNT purity is crucial in achieving optimal material properties [83,135]. The final product is CNTs like those shown in the AFM images in Figure 9c [135].

Once the CNTs have been synthesized, dispersing them in printable ink is a key challenge [92]. In designing a screen printable CNT ink, Menon et al. dispersed CNTs in an ethanol SDS solution optimized to 7.5 wt.%, then added various PVP loadings and assessed printability [135]. It was determined that PVP weights equal to half that of the CNTs were most suited for screen printing [135]. In addition, Shi et al. demonstrated that sonication is crucial in SDS facilitated CNT dispersion because the sonication forcibly breaks apart CNT clusters, exposing the CNT surface to SDS [87]. Figure 9d depicts TEM image results of one such experiment, where dispersion clearly improves after sonicating for 6 instead of 4 h [87]. Gravure printed semiconducting SWCNTs have been thoroughly studied for thin-film transistor applications, and Sun et al. recently demonstrated a thin film transistor active-matrix (TFT-AM) electrophoretic sheet on PET that could be used as a wearable display [136]. Metallic CNTs were removed from a mixed semiconducting-metal powder with poly(9,9-didodecylfluorene) (PFDD) in a PFDD/CNT ratio of 1.25/1.00 to yield a semiconducting purity of 99.9%. The PFDD was then exchanged with a polythiophene derivative (P3ME4MT) in toluene and dispersed in 1-octanol to produce a printable viscosity and suitable capillary number. After gravure printing at 6 m/min at 30 µm depth and 150 µm cell opening for 10 PPI resolution and 10 µm depth and 35 µm opening for 40 PPI, TFT-AMs with average mobility of 0.23 ± 0.12 cm^2^ V^−1^ s^−1^, the average on-off ratio of 104.1, and threshold voltage variation of ±13% was demonstrated [136]. Images of the printed TFT-AM are provided in Figure 9d [136]. Finally, inkjet printing of CNTs has been demonstrated for numerous biosensor, conductor, and semiconductor applications [137]. For instance, Okimoto et al. improved on previous CNT semiconductor performances by optimizing the CNT density in a novel SWCNT inkjet printing ink [138]. The SWCNTs were prepared by laser vaporizing carbon rods doped with Co/Ni in an argon environment and purified with H_2_O_2_, HCl, and NaOH. The SWCNTs were dispersed in DMF in a mixture of 0.04 µg/mL, sonicated, centrifuged, and filtered through poly(tetrafluoroethylene) membrane filters. The ink was printable with a 30 µm nozzle at 500 Hz, and the fabricated CNT TFT yielded mobility of 1.6 to 4.2 cm^2^ V^−1^ s^−1^ and an on/off ratio of 4–5 digits [138]. Because CNT inks are both highly desirable for commercial sensing and TFT applications and the large challenges in designing printable inks, numerous commercial inks are now available, and many reported works in the literature are using these inks for inkjet and gravure printing (Figure 9e) [91,92]. In summary, CNT ink printing is highly attractive for many essential bioelectronics’ applications, and new continued investigations into high throughput fabrication methods are essential in translating these novel discoveries to industrial and clinical use.

#### 3.5.2. Post Print Processing

Post print processing for CNT inks varies greatly based on the specific dispersants and polymers employed in the ink synthesis [136,139]. Generally, the processing is complex, which creates an incentive to remove as much of the polymer residue and dispersant before printing as possible [135]. For instance, sonication, centrifugation, washing and filtering before printing are typically essential measures to create an environment in which post-print processing is feasible [87]. In addition, careful selection of polymers and dispersants and effective processing can be employed to yield simple and effective processing [92,140]. Although several complicated polymer removal strategies have been studied, such as metal–chelation-assisted polymer removal (McAPR) and yttrium oxide coating, washing and annealing are still the most preferred because of simplicity, cost, and scalability [91]. For example, Yu et al. recently removed polycarbazole (PCz) from a CNT print via THF washing [140]. Although some PCz remained in ink, this method is an effective and simple mechanism for biocompatible post-print CNT processing [140]. Another common washing solvent is toluene which is often used with elevated temperatures to improve solubility. Annealing is also highly effective, but high-temperature restraints (above 300 °C) make it not suited for many flexible substrates, such as PET and TPU [83]. In a modified annealing process, one may exchange the polymer for a different material, and this process is both effective and suited to lower temperatures [139]. For instance, Sun et al. exchanged PFDD for P_3_ME_4_MT, as discussed previously, to create a high mobility electrophoretic deposition [136]. Overall, post-print processing for CNT inks is an area of high research interest, and novel advancements are greatly needed to implement CNT imprinting in high throughput fabrication processes fully.

### 3.6. Novel 2D Nanomaterials

One of the critical advantages of nanomaterial printing is the opportunity to tune material properties to address various application needs finely. The development of novel 2D materials is essential in the high throughput printing of advanced biosensors and bioelectronics [141]. For instance, two-dimensional transition metal dichalcogenides (TMDs), such as WSe_2_, WS_2_, MoSe_2_, and MoS_2_, are direct bandgap monolayers with high flexibility that can be used alone or in combination with graphene to create various flexible sensors [141,142,143,144]. TMDs have several exceptional material properties that make them highly suited for many electronics applications. They contain no inversion center, which allows the k-valley index to be manifest as a new degree of freedom charge carrier [144]. Strong spin-orbit coupling leads to spin-orbit splitting, making them well suited to spin transport electronics applications, commonly termed spintronics [145]. Printable TMD inks can be synthesized from bulked cellular samples through liquid-phase exfoliation (LPE). This has been demonstrated for applications such as screen-printed oxygen sensing electrodes [42] and wearable heterostructure photodetectors [143]. Despite recent advances in LPE processes by optimizing dispersion agent concentrations, polymers, stabilizers, and binders, TMDs can be challenging to disperse in printable inks. Many LPE processes still rely on toxic and hazardous materials that do not demonstrate biocompatibility for wearable applications [146]. Lee et al. developed a zwitterion-assisted LPE process to synthesize TMDs in water to address this concern, allowing for the development of highly-biocompatible TMD inks [146].

Additionally, hexagonal boron nitride (h-BN) is a high bandgap, biocompatible, nanomaterial isostructural to graphene that is highly suited for nanophotonics. It is a natural hyperbolic material in the mid-IR range [147], attractive for use as a substrate for graphene transistors because of its atomic-scale smoothness [32], advantageous for electrochemical sensing [148], of great interest as a capacitive dielectric [130], and potentially suited for the in-situ formation of 1D conducting channels [57]. Printable h-BN monolayers may be synthesized through top-down approaches, such as mechanical and chemical exfoliation, or bottom-down approaches, such as PVD and CVD [148]. Because h-BN has strong in-plane covalent bonds and weak inter-plane van der Waals forces compared to graphene, h-BN is an attractive 2D material for printable inks. Although h-BN has long been of interest, its potential for high-throughput fabrication via screen and inkjet printing has just recently been appreciated [148]. For instance, h-BN is now well understood for capacitive, dielectric, and transistor substrate applications. Still, new investigations into printable optic devices and electrochemical sensors will be needed to unlock this material’s full potential. In one recent work, Desai et al. optimized h-BN nanoplatelet geometries synthesized through exfoliation and deposition thicknesses to yield a printed photo-capacitor with excellent thermal stability ranging from 6–350 K [130]. Additionally, Angizi et al. used edge functionalized h-BN dispersed in ethanol for screen printed Vitamin C detection in a flexible biosensor [149].

## 4. Applications for Bioelectronics

### 4.1. Electrical Interconnections

Conductive interconnections are the backbone of all fully integrated electronic devices [10]. These traces form the basis of circuits, and they must exhibit high conductivity and reliability. Furthermore, many applications require the circuit to stretch and deform [8]. For instance, a skin-mounted electrophysiology sensor is highly degraded by motion artifacts, and a stretchable circuit can greatly reduce these artifacts [4,17]. Interconnections for these soft, flexible, and stretchable devices have followed a three-stage development process: first came the development of stretchable interconnections based on fractal geometries fabricated with traditional MEMS processes [10,150]. Second, recent works have sought to fully print these systems on non-conventional substrates, such as TPU and PET, that are not compatible with MEMS fabrication [150]. Finally, these printed methods are being scaled with high throughput methods to make them suitable for commercial scales.

Several key challenges must be overcome in this third stage of interconnection fabrication. Crucially, they must be printed with high resolutions, speed, conductivity, and reliability on a variety of non-traditional substrates, and many applications require high resistive stability with local strain [9,10,17]. Although local strain can be alleviated with optimized geometries, printed interconnects are often embedded in a polymer matrix, which allows them to form conductive networks that remain conductive with strain [11,35,64]. These interconnections are typically stretchable up to 10% for wearable applications, although in some cases, stretchability up to 100% has been demonstrated [151]. However, the addition of polymer matrices often limits interconnect conductivity, which can often approach the limit set by bulk metals when sintered on temperature-stable substrates. As mentioned previously, these thin films are made flexible despite their high modulus through thin deposition heights, and they can stretch as a system without high local strain through optimized geometries [35]. However, these geometries may require spatial resolutions approaching the limits of fully printed technologies [152].

**Figure 10:**
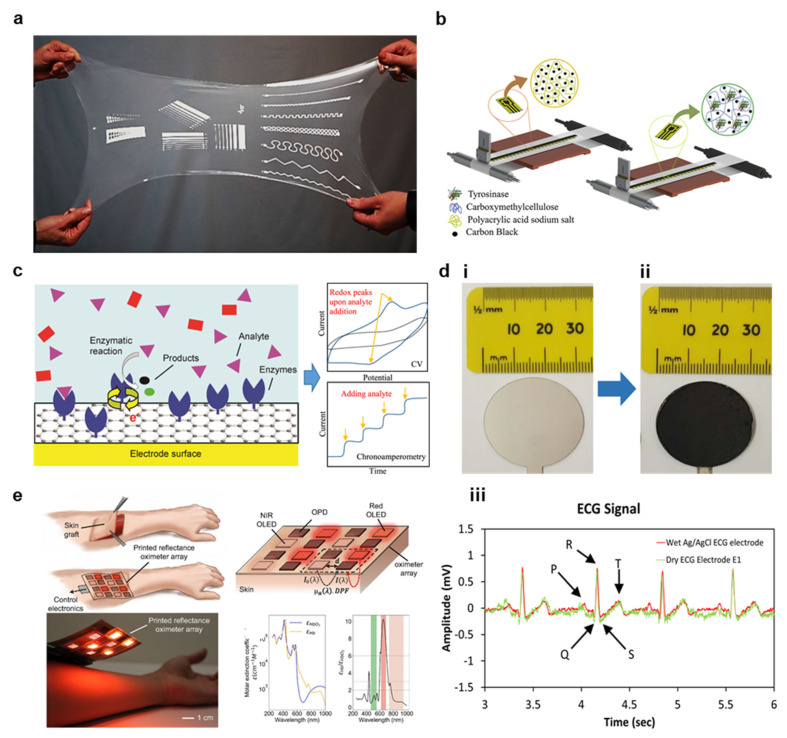
Bioelectronics applications (**a**) Image of screen-printed conductive traces. (reprinted with permission from *Ind. Eng. Chem. Res*. (2019), 58, 43, 19909–19916, Copyright 2020, ACS). (**b**) Illustration of roll-to-roll slot-die coated electrochemical sensors. (reprinted with permission from *Biosens. Bioelectron.* (2020), 165, 112428. Copyright 2020 Elsevier). (**c**) Schematic of a graphene-based enzymatic biosensor. (reprinted with permission from *Biosens. Bioelectron.* (2017), 87, 7–17. Copyright 2017 Elsevier). (**d**) Screen-printed ECG electrodes with (**i**) Ag and (**ii**) MWCNT layers. (**iii**) Example ECG signals are shown compared to commercial Ag/AgCl gel electrodes. (reprinted with permission under Creative Commons license CC BY-NC-ND 4.0 from *Sens Biosensing Res* (2018), 20, 9–15.) (**e**) Overview and operation of a screen-printed reflectance oximeter array (ROA). (**Top left**) placement of the ROA after skin graft surgery. (**Top right**) illustration of ROA pixel array. (**Bottom left**) Image of the printed ROA. (**Bottom right**) Molar extinction coefficients for oxygenated and deoxygenated hemoglobin as a function of wavelength. (reprinted with permission from *PNAS*, (2018) 115 (47) E11015–E11024, Copyright 2018, PNAS).

A summary of recently reported interconnections fabricated with high throughput processing is provided in Table 2, showing the different substrates, materials, fabrication methods, and curing approaches employed in state-of-the-art processes. In addition, resistances and resolutions are compared for each system, indicating which methods are preferred for each specific use case and application. When high conductivity is required, NPs and NWs inks with high material loadings are preferred, and sintering is often required in the case of NPs [17,153,154]. However, Scheideler et al. and Ohsawa et al. were able to achieve high conductivities on polyethylene naphthalate (PEN) substrates using NWs and NPs, respectively, without sintering. In addition to conductivity, the inks should not be significantly higher modulus when cured than the substrate, or advanced geometries are needed to alleviate local strain [35]. With optimized ink compositions and judicious trace patterning, very high reliability during bending, washing, and other wearable use can be demonstrated [82]. Finally, the same geometries that are effective in strain relief for high modulus MEMS interconnects are not always the ideal choice for stretchable interconnects because of the complex mechanics introduced when the substrate itself stretches and deforms from Poisson effects and the substrate-ink modulus mismatch [35]. Therefore, Huttunen et al. performed an experiment to assess different trace geometries of AgNP inks on a PDMS substrate, determining that triangular patterns maintained conductivity with higher applied strain [35]. An example of several patterns printed on a stretchable PDMS sheet is provided in Figure 10a [35]. In summary, recent developments in high throughput interconnect printing allow one to produce patterns with conductivity and stretchability optimized to many bioelectronic applications. Still, more thorough testing and process optimization are required for these systems to become commercially adopted.

### 4.2. Biosensors

Biomolecule sensing devices, such as glucose-sensing patches, promise a transformative way to continuously assess crucial biomarkers, making their development critical in the future of healthcare development [12,14,15,16]. Traditional biosensing methods, as discussed in the introduction, are highly limited because of exceptional costs and the inability to record results continuously in real-time, whereas printed biosensors are well suited to long-term, continuous monitoring in an affordable and wearable package [12,13]. The clinical implication of such technology is clear, and the development of high throughput biosensor fabrication, such as the slot die process that is shown in Figure 10b [57] It is of very high importance [13]. Generally, biosensors consist of a receptor, e.g., an antibody, and transducer, e.g., a nanomaterial sheet capable of transmitting an electrical signal from the receptor to a circuit element [13,16]. To bind the receptor to the transducer, the material must be functionalized, as discussed in Section 3.4. Although many nanomaterials may be effectively functionalized, graphene is one of the most favorable materials because of the ease in which one can attach a variety of organic and inorganic functional groups [13]. For instance, graphene can be oxidized, then functionalized with 1-ethyl-3-(3-dimethylaminopropyl) carbodiimide hydrochloride (EDC)/N-hydroxysuccinimide (NHS) (EDC/NHS) to facilitate antibody binding [156]. The target molecule detection can be achieved through several methods, although electrochemistry is the most employed [12,13]. An example of a potentiometric electrochemistry analysis is provided in Figure 10c [12]. In these systems, functionalized working (where the reaction occurs) and reference (where the current is provided) electrodes are implemented, and the transducer is able to record changes in current, resistance, or potentially caused during the binding reaction [13]. Another printed biosensor method is based on FET technology, where the binding of a target molecule is used to modulate the flow of current through a channel [13].

Table 3 summarizes recent demonstrations of high throughput biosensor fabrication, with an emphasis on device performance [16,40,157,158,159,160]. Although each work incorporated high throughput fabrication methods, many did not specifically state key process parameters required to translate this technology, such as print speed, roll pressure, ink viscosity, and in some cases, curing [12]. Instead, these works focused primarily on device efficacy, likely because there are significant unanswered questions in biosensor printing relating to material choice and functionalization. However, Cagnani et al. were able to achieve an exceptionally high 30 m/min printing speed using a slot die coating on PET for dopamine detection [151]. Bariya et al. developed a high throughput gravure printing method for wearable sweat sensor fabrication capable of 6 m/min printing [40]. The majority of additional works focused primarily on sensor stability, which itself is highly dependent on material functionalization, purity, and receptor choice. For instance, Narakathu et al. used gravure printing to fabricate AuNP electrodes for the detection of a variety of chemicals, such as mercury sulfide (HgS), lead sulfide (PbS), D-proline, and sarcosine, demonstrating high sensitivity down to pico-molar concentrations [158]. In another experiment, Favero et al. demonstrated that graphene and MWCNT functionalized electrodes can be improved with the ingrafting of AuNPs to increase conductivity, noticing a >10% increase in electroactive area. A corresponding increase in R correlations indicates linearity after the addition of AuNPs [157]. Although biosensor printing is an active area of research with significant hurdles to overcome, the study of high throughput fabrication methods for the sensors that have been well tested, such as glucose and sweat monitors, is highly needed to scale these methods into clinical practice.

### 4.3. Additional Applications

There is a great diversity of potential bioelectronics applications, and this review will focus on those that have gained attention for high throughput fabrication. However, many other applications, such as implantable cerebrovascular and arterial stents, brain–machine interfaces, and fully printed wearable devices, are of tremendous interest [10,150]. Other systems have been successfully fabricated with high throughput methods, as summarized in Table 4 [37,129,162,163,164,165,166]. One area of critical interest is wearable electrophysiology monitoring. Traditional electrocardiogram (ECG), electromyogram (EMG), electrooculogram (EOG), and electroencephalogram (EEG) electrodes are based on a hydrogel that can cause irritations in long term use, especially in neonates and those with sensitive skin, and they are highly prone to motion artifacts [8,17]. In contrast, printed dry electrodes can conform to the patient’s skin and interface without any damaging gels, making them excellently suited to continuous monitoring, even during patient motion [9,10]. In two reported works, Tan et al. and Chalihawi et al. used carbon black and AgNP and MWCNTs, respectively, to fabricate dry electrodes. Tan et al. used doctor blade coating on a TPU substrate to produce high-performing electrodes for textile integration that can endure over 50 washing cycles [165]. In addition, Chalihawi et al. screen printed AgNP interconnections and an electrode pad, then used doctor blade coating to deposit a functional MWCNT sensor, which was shown to achieve similar ECG signals when compared to a gel-based Ag/AgCl sensor [166]. Images of the fabricated electrodes with (i) Ag layer and (ii) MWCNT layer are provided in Figure 10d, and the ECG performance is shown in (iii) [166]. Although it was not assessed, it would be of great interest to determine these electrode’s performance during patient motion. Another interesting area of research is the development of capacitive touch sensors, which have been widely reported in the literature using traditional MEMS fabrication. Lee et al. created such a touch sensor with an air gap instead of PDMS dielectric, and noted that the increased dielectric constant of air allowed for highly improved sensitivity (ΔC/C_0_ (%) of 0.118%) and high linear sensing range from 0–20 KPa [37]. One area of high interest is in printing on TPU substrates, and this was the focus of a recent investigation by Jansson et al. using screen-printed AgNP inks. In this experiment, various dimensions were cut in a roll-to-roll laser process and filled with AgNP inks, and ink was filled from both the cutting side and the opposite side [162]. It was determined that the via diameter had a minor impact on conductivity and reliability, but the match between via diameter and screen opening, optimization of printing thickness and side from which the via is printing were of high importance [162]. In addition, Alsuradi et al. demonstrated a very high control of capacitive and inductive behavior in screen-printed traces based on geometries adapted from integrated microwave circuits, then optimized for thicker depositions common in screen printing [129]. As a result, inductances and capacitances could be reliably controlled to within 5% error, which is considered acceptable for many commercial passive components [129]. Finally, polymer materials like poly(3,4-ethylenedioxythiophene) polystyrene sulfonate (PEDOT:PSS) are outside the scope of this review, but it is worth noting that polymer inks can be printed with high throughput methods, such as screen printing, to manufacture bioelectronics systems. For instance, Khan et al. demonstrated a fully printed PEDOT:PSS photoplethysmography array based on screen printing for use in patients recovering from skin graft surgery. The device is shown along with sensitivities to oxygenated and deoxygenated hemoglobin in Figure 10e [163]. In summary, many additional bioelectronics applications could be scaled with high throughput fabrication methods. It is an open challenge to the reader to apply these techniques to their area of expertise.

## 5. Conclusions and Future Outlook

Recently, various high-throughput nanomaterial fabrication methods have been demonstrated for hybrid bioelectronics. But, there remain substantial challenges to be overcome. Recent attempts to optimize printing parameters for gravure, flexography, screen, inkjet, and slot die printing have opened new possibilities for highly scalable soft electronics and critically needed hybrid biosensors. In addition, novel approaches to high throughput printing, such as flexography aided by CNT stamps, set the leading edge in print resolution, homogeneity, and quality [60]. Significant progress within the last decade on ink rheology optimization and material-interface studies allows for the high-resolution patterning of many functional nanomaterials. And these materials are being extensively studied for a diverse set of bioelectronics applications. This field, however, remains in its infancy, and there are several critical challenges to be overcome. First, electronic circuits require more than interconnects, and the further study of printed vias and material adhesion in multiple layer prints is of high importance. Second, printing resolutions remain low for many methods; thus, new approaches to increasing resolution must be investigated. Third, inkjet printing offers substantial advantages in prototyping and manufacturing costs. Still, recent works have not implemented commercially tested roll-to-roll inkjet printing to the degree necessary to make inkjet printing a desirable method for high throughput nanomaterial fabrication. Finally, such as gravure roll geometries and materials, printing parameters should be reimagined for nanomaterial applications instead of simply relying on processes optimized for inks without large-volume loadings or dispersion challenges. Overall, recent progress in the field of nanomaterial printing offers great hope that a new class of hybrid flexible bioelectronics can provide the affordable, long-term usable devices necessary to help the millions of people suffering from undiagnosed diseases. However, new investigations into novel printing approaches and further optimization of current methods are greatly needed before this vision is made a reality.

## Figures and Tables

**Table 1:** Summary of nanomaterial fabrication methods with key advantages and limitations.

Printing Method	Typical Min Resolution [µm]	Printing Speed [m/min]	Ink Viscosities [mPa.s]	Ink Surface Tension [mN/m]	Pros	Cons
Gravure	30	1–20	100–500	20–40	High speed [40] Good reliability [27] Long production runs [27]	High startup costs [27] Expensive prototyping [27]
Flexography	30	1–20	50–500	10–30	High Speed [41] Easier to prototype than gravure [29]	High startup costs [29] Lower durability than gravure [41]
Screen	50	0.1–15	500–10,000	35–50	Inexpensive to prototype [42] Balance between speed, reliability, and cost [35] Simple process optimization [43]	Limited resolution [24] Strict ink rheology requirements [44]
Inkjet	30	0.01–15	1–20	10–30	No additional cost to prototype [45] Excellent resolution and pattern control [46]	Complicated to integrate with roll–to–roll systems Nozzle clogging [47] Coffee ring effect [48]
Slot die	40	1–50	2–500	–	Efficient and precise coating of homogeneous films [30]	Not suited for complex patterning [49]

**Table 2:** High-throughput nanomaterial interconnection fabrication.

Reference	Material	Method	Substrate	Curing	Printing Speed [m/min]	Sheet Resistance [Ω/sq]	Resolution [µm]
[53]	Graphene	Gravure	PI	Room temperature	0.3	6.25	30
[22]	AgNP	Inkjet	PEN	Laser Sintering	10	2.5	50
[155]	AgNP	Flexography	PET	130 °C for 5 min	5	45	150
[35]	AgNP	Screen	PDMS	140 °C for 8 min	2	2.5	125
[50]	AgNW	Gravure	PET	150 °C for 5 min	1.5 mm/s	~20	50–150
[153]	AgNW	Screen printing	PET	Flash Light Sintering	0.2	9.6	20
[154]	AgNW	Gravure	PEN	170 °C for 10 min	1	9.3	Film was tested
[88]	AgNP	Gravure	PEN	100 °C for 1 min	Not reported, roll-to-roll	4.9	40 mm × 80 mm

**Table 3:** High-throughput nanomaterial-based biosensor fabrication.

Reference	Material	Method	Substrate	Curing	Printing Speed [m/min]	Application	Reported Efficacy
[161]	Carbon	Slot Die	PET	60 °C for 2 min	30	Dopamine detection	Sensitivity of 0.32 µA L/μmol with limit of detection (LOD) of 0.09 μmol/L
[160]	AgNP	Ink-jet	PET	No Post-print treatment	-	Antibiotic detection in milk	100–10,000 μg/mL with LOD of 10 μg/mL
[40]	AgNP	Gravure	Paper	120 °C for 2 min	6	Sweat sensing	Error of 1.4% over a range of ~4–100 [Na^+^] (mM)
[159]	AgNP	Gravure	PET	Not reported	Not reported	IgG sensing	2–5% sensitivity to IgG over 10 pM-10 μM concentrations
[158]	AgNP	Gravure	PET	Not reported	Not reported	Sacrosine sensing	Resistance changed from 299 Ω to 325 Ω with varying concentration from 1 pM to 100 mM
[16]	Graphene	Screen printing	Textile	Not reported	Not reported	Influenza sensing	Stabile sensing over 10 ng/mL to 10 μg/mL with a limit of detection of 10 ng/mL.
[157]	AuNPs and MWCNTs	Screen printing	Glass	Not reported	Not reported	Laccase sensing	Linear range of 1–100 μM with a LOD = 0.5 μM and a sensitivity of 0.051 μA·μM^−1^

**Table 4:** Additional bioelectronics applications fabricated with high throughput methods.

Reference	Material	Method	Substrate	Curing	Printing Speed [m/min]	Application	Reported Efficacy
[165]	Carbon Black	Doctor Blade Coating	TPU	Belt fed convection oven at 80 °C	Not reported	ECG monitoring	Quality signals after 50 wash cycles.
[166]	AgNP and MWCNT	Screen print AgNP then Doctor blade MWCNTs	PET	120 °C for 20 min	Not reported	ECG monitoring	Equivalent ECG signals to gel Ag/AgCl comparison
[37]	AgNP	Slot Die	PET	150 °C for 10 min.	0.5	Cantilever touch sensor	ΔC/C_0_ (%) of 0.118% for the range 0–20 kPa
[162]	AgNP	Screen printing	TPU	140 °C for 2 min.	2	Via Filling	Low (10–40Ω resistance over the range 0–100 stretching cycles with <10% strain
[164]	AgNP	Screen printing	TPU	Not reported	Not reported	Oxygen sensing	0.1 ppm of O_2_ sensitivity with 40 PPM LOD
[129]	AgNP	Screen printing	hydrocolloid dressings	Not reported	Not reported	Passive elements (e.g., capacitors)	Control of impedance and capacitance within 5% error
[163]	PEDOT:PSS	Blade Coating	PEN	120 °C for 10 min.	0.6	Photoplethysmography (PPG) array for SpO_2_ monitoring	Mean error of 1.1% compared to a commercial device

## Data Availability

No new data were created or analyzed in this study.
